# Proteomics insight into phosphate limitation response in Burkholderia phytopathogens

**DOI:** 10.1099/mic.0.001758

**Published:** 2026-10-15

**Authors:** Lixian Xu, Manpreet Kaur, Jong Hyun Ham, Mengcen Wang, Robert W. Jackson, Yin Chen

**Affiliations:** 1 School of Biosciences, University of Birmingham, Birmingham, B15 2TT, UK; 2 Institute of Microbiology and Infection, University of Birmingham, Birmingham B15 2TT, UK; 3 Department of Plant Pathology and Crop Physiology, Louisiana State University Agricultural Center, Baton Rouge, LA 70803, USA; 4 College of Agriculture and Biotechnology, Zhejiang University, Hangzhou, Zhejiang 310058, PR China

**Keywords:** *Burkholderia *spp.,, lipid remodelling, phytopathogens, phosphorus stress response

## Abstract

Inorganic phosphate (Pi) limitation is a pervasive environmental constraint that shapes microbial behaviour in soil and plant-associated niches. To determine how Pi scarcity influences the pathogenic potential of rice-infecting *Burkholderia* species, we performed comparative proteomic and lipidomic analyses of *Burkholderia glumae* and *Burkholderia plantarii* grown under Pi-replete and Pi-deplete conditions. Pi starvation triggered strong activation of the Pho regulon in both organisms, including induction of alkaline phosphatases, phosphonate transporters and the high-affinity PstSABC uptake system. Beyond classical phosphate-stress responses, Pi limitation markedly enriched proteins associated with virulence, secretion systems, toxins, chemotaxis and cell envelope modification. Both species also activated PlcP/Agt-mediated lipid remodelling pathways and synthesized phosphorus-free glycolipids exclusively during Pi depletion, indicating substantial membrane lipid restructuring. These results demonstrate that Pi limitation acts as a major environmental cue coordinating nutrient acquisition with multiple virulence-related functions. Our findings provide new insight into how phosphate scarcity shapes the environmental fitness and pathogenic capacity of *Burkholderia* phytopathogens and highlight nutrient limitation as an important driver of plant disease emergence.

## Data Availability

The data that supports the findings of this study are available in the supplementary material of this article.

## Introduction

The *Burkholderia* genus was established in 1992 and includes strains that infect humans, animals and plants [1, 2]. Within this genus, three rice-infecting strains have been identified, which are *Burkholderia plantarii*, *Burkholderia glumae* and *Burkholderia gladioli*. Both *B. plantarii* and *B. glumae* are motile phytopathogens that thrive in warm, humid environments, including soil, typical of tropical and subtropical rice-growing regions. *B. glumae* is the causative agent of bacterial panicle blight which poses a significant threat to global rice production [3–5]. *B. plantarii* causes rice seedling blight, leading to significant agricultural losses in tropical regions [6]. Their virulence is mainly attributed to their ability to produce phytotoxins. However, other factors, such as quorum sensing systems, secretion systems and their ability to produce lipases also contribute significantly to their virulence [3, 4, 6–8]. Due to climate change and rise in global temperatures, the increased occurrence of *Burkholderia* phytopathogens in soil and water poses a serious threat to global food production [9]. Furthermore, species of the *Burkholderia* genus are capable of causing disease in humans, the incidence of which may also rise with an increase in their global spread [10]. Therefore, understanding the biology of this genus is important for understanding different host interactions.

Phosphorus is a critical nutrient for microbial growth. Phosphorus is involved in energy metabolism, photosynthesis, cell division and the synthesis of key biological macromolecules. Compared with nitrogen, inorganic phosphate (Pi) in soil comes from a single source of rock phosphate and is easily lost, so soil bacteria are prone to Pi deficiency [11]. One strategy that bacteria have developed to obtain more phosphorus is by exploiting host plant cells. When *Arabidopsis* plants grown in Pi-limited soil are exposed to a bacterial synthetic community (SynCom) composed of 185 members, bacteria from *Burkholderia* genus are enriched in the plant tissues [12]. Furthermore, plant shoots accumulate more orthophosphate if they are infected with SynCom without *Burkholderia* than with full SynCom, in Pi-limited conditions. However, the mechanistic understanding of survival strategies adopted by *Burkholderia* phytopathogens under Pi-limited conditions is very incomplete.

Apart from exploiting the host, bacteria use a variety of other molecular and cellular mechanisms to enhance their chance of survival in Pi-limited environments. In Gram-negative bacteria, the PhoBR two-component system (TCS), which consists of the histidine kinase PhoR and its cognate DNA-binding response regulator PhoB, plays a crucial role [13, 14]. However, since PhoR lacks the periplasmic sensing domain, it depends on the phosphate transporter PstSABC, a member of the ATP-binding cassette (ABC) transporter superfamily, to coordinate the sensing of cellular Pi levels [15–17]. A model has been suggested in which PstSABC adopts different conformational states depending on whether it is transporting Pi into the cell or not [18]. When the extracellular Pi levels are high, the phosphate-loaded dikinase state after which PhoR phosphorylates the receiver domain of PhoB. Phosphorylated PhoB binds to a specific DNA sequence called PHO box, which is present in the promoter region of all the genes that are activated by PhoB and are collectively referred to as Pho regulon [19, 20]. The interaction between PstSABC and PhoR is also mediated by PhoU which reduces the transport rate of PstSABC under high Pi conditions [21, 22].

Some of the components which are part of the Pho regulon include phosphatases, which hydrolyse phosphoesters to release Pi, *phn* genes that are involved in the transport and degradation of phosphonates and genes involved in polyphosphate synthesis [23, 24]. PhoB also activates *plcP* that encodes for a phospholipase and *agt* that encodes for a glycosyltransferase [25, 26]. PlcP hydrolyses membrane phospholipids, such as phosphatidylethanolamine (PE), phosphatidylglycerol (PG) or phosphatidic acid (PA) into diacylglycerol (DAG), whereas Agt adds a sugar residue to DAG, enabling the biosynthesis of phosphorus-free lipids. These include a variety of non-P lipids, such as betaine lipid diacylglyceryl trimethylhomoserine, glycolipids monoglucosyl diacylglycerol (MGDG) and glucuronic acid diacylglycerol (GADG) [27]. Lipid remodelling has been studied in a limited number of bacteria where it has been linked to virulence and antimicrobial resistance. In *Pseudomonas aeruginosa*, phosphate limitation induces PlcP-mediated phospholipid degradation and drives the synthesis of novel phosphate-free glycolipids. These glycolipids are integrated into the outer membrane, reducing its permeability and thereby enhancing bacterial resistance to the cationic antimicrobial peptide polymyxin [28]. In *Burkholderia cenocepacia*, a *plcP* mutant, which is unable to remodel its phospholipids, was found to be significantly less virulent than the WT strain in a *Galleria mellonella* infection model [25].

In this study, we analysed the proteome of *Burkholderia* phytopathogens *B. glumae* and *B. plantarii* cultured in the absence and presence of Pi. We found many proteins which were either significantly enriched or depleted in −Pi condition as compared to the +Pi condition in both strains. Among the significantly enriched proteins, those involved in phosphate stress were identified, as expected. Furthermore, proteins related to antimicrobial resistance, lipid remodelling, LPS biosynthesis, bacterial toxins, chemotaxis, secretion systems and virulence were also significantly enriched in −Pi condition. This study, therefore, provides a unique insight into the association between phosphate stress and pathogenesis in *Burkholderia* phytopathogens.

## Methods

### Bacterial strains and culture conditions

*B. plantarii* strain ATCC 43733 was sourced from the DSMZ culture collection and *B. glumae* strain 336gr-1 was maintained at Louisiana State University, USA. 15% (v/v) glycerol stocks in LB were prepared for both the strains and stored at −80 °C. The strains were retrieved from glycerol stocks by plating out on LB agar (LB; 1% wt/v tryptone, 0.5% wt/v yeast extract, 1% wt/v NaCl+1.5% (wt/v) agar; pH 7) and incubating at 30 °C for 2–3 days. For pre-cultures, single colonies were inoculated into 5 ml LB broth and grown overnight (30 °C, 200 r.p.m.). Secondary cultures were initiated by transferring 150 µl of pre-culture into 50 ml fresh LB broth and incubating under identical conditions.

### Phosphate starvation and alkaline phosphatase assay

Bacteria were grown in LB medium overnight for 18–20 h so that the OD_600_ of the culture medium was above 2. Next day, overnight cultures were collected by centrifugation (4,000 r.p.m., 15 min, 4 °C) and washed twice with phosphate-free minimal medium A (MMA) which constitutes Na-Succinate 5.4 g l^−1^, NaCl 200 mg l^−1^, NH_4_Cl 450 mg l^−1^, CaCl_2_ 200 mg l^−1^, KCl 200 mg l^−1^, MgCl_2_ 450 mg l^−1^, FeCl_2_10 mg l^−1^, MnCl_2_ 10 mg l^−1^, 10 mM HEPES buffer at pH 7 and 0.01% (wt/v) yeast extract. Cells were finally resuspended in MMA supplemented with either 0 mM (phosphate-deplete) or 1 mM (phosphate-replete control) Na_2_HPO_4_. After resuspension in MMA, the OD_600_ of the cultures was measured at 0 and 24 h timepoints. To ensure that the cells were phosphate-starved, alkaline phosphatase activity of the cultures was measured at 0 and 24 h before collecting samples for proteomics and lipidomics. This was achieved by mixing 450 µl of the culture aliquot with either 50 µl of Tris-HCl (pH 8) at 10 mM final concentration or 50 µl of *p*-nitrophenol phosphate at 1 mM final concentration. Reactions were incubated in darkness for 1 h at room temperature (~22 °C) before being centrifuged (13,000 r.p.m., 10 min). The absorbance of the supernatant containing *p*-nitrophenol (pNP) was measured at 405 nm using a Tecan Spark^®^ Cyto plate reader. pNP concentrations were calculated using a calibration curve. Three biological replicates of the experiment were performed. Statistical analysis was performed using GraphPad Prism 10, version 10.2.3, downloadable from: https://www.graphpad.com/.

### Protein extraction and digestion

The strains were cultured in triplicates in MMA, either in the absence or presence of 1 mM phosphate (Na_2_HPO_4_), for 24 h before samples were collected for proteomics by centrifugation (4,000 r.p.m., 15 min, 4 °C). The isolation of peptides from bacterial cells was carried out as described previously [29]. Briefly, the cell pellets were resuspended in 3 : 1 water: LDS buffer (1% β-mercaptoethanol, v/v). Cells were then lysed at 95 °C for 5 min. Approximately 20 µl of sample was then run on a precast SDS-PAGE NuPAGE gel for 5 min to remove cell debris before Coomassie staining. After staining, each protein band was excised, de-stained with 50% (v/v) ethanol/50 mM ammonium bicarbonate (55 °C, 650 r.p.m.), dehydrated in 100% ethanol and reduced with 180 µl of Tris(2-carboxyethyl)phosphine (70 °C, 5 min). Gel pieces were digested with trypsin (37 °C, overnight) in 50 mM ammonium bicarbonate. Peptides were extracted via sequential sonication in 25% (v/v) acetonitrile/5% (v/v) formic acid, concentrated using a centrifugal evaporator (45–60 °C, 2 h) and filtered through cellulose acetate membranes (5,000 r.p.m., 5 min). Extracts were stored at −20 °C prior to LC-MS/MS analysis.

### Proteomic analysis using LC-MS/MS

Reversed-phase chromatography was used to separate tryptic peptides prior to mass spectrometric analysis. Two C_18_ columns were utilized: an Acclaim PepMap µ-precolumn cartridge 300 µm i.d.×5 mm, 5 µm, 100 Å (Thermo Fisher Scientific) and a 75 µm×25 cm, 1.5 µm (Bruker PepSep ULTRA Analytical column). The Ultimate 3000 RSLCnano system (Thermo Fisher Scientific) was used with mobile phase solvent A composed of 0.1% formic acid in water and mobile phase solvent B composed of 0.1% formic acid in acetonitrile. Samples were loaded on the µ-precolumn equilibrated in 2% aqueous acetonitrile containing 0.1% trifluoroacetic and peptides were eluted onto the analytical column at 300 nl min^−1^ by increasing the concentration of mobile phase solvent B from 4 B to 25% over 36 min, then to 35% B over 10 min and to 90% B over 3 min, followed by a 10 min re-equilibration at 4% B.

Ultimate 3000 RSLCnano was coupled online to a hybrid trapped ion mobility spectrometry with quadrupole time-of-flight (TIMS-TOF) Pro (Bruker Daltonics, Germany) via a CaptiveSpray nano-electrospray ion source [30]. The TIMS-TOF Pro was operated in Data-Dependent Parallel Accumulation-Serial Fragmentation mode. Peptides were separated by ion mobility depending on their collisional cross sections and charge states. The method settings were as follows: mass range 100 to 1,700 m/z, ion mobility range 1 /K0 Start 0.6 Vs/cm^2^ End 1.6 Vs/cm^2^, Ramp rate 9.42 Hz and Duty cycle 100%.

### Proteomics MS-MS data analysis using MaxQuant and Perseus

The raw MS/MS data obtained from the experiment were first analysed using MaxQuant software, version 2.5.2.0. This platform enables automated processing of MS/MS data, including protein identification and quantification based on label-free quantification and data normalization. The output files generated by MaxQuant were then imported into Perseus software, version 2.0.11.0 for statistical and bioinformatic analysis. In Perseus, the data were subjected to missing value filtering and imputation, log_2_ transformation, differential expression analysis (such as *t*-tests or ANOVA), as well as clustering and principal component analysis (PCA). Proteins with significantly altered abundance (*P*<0.05) in −Pi condition as compared to the +Pi condition and |log_2_FC|>1 or < -1 were assigned to different KEGG BRITE categories using BlastKOALA, version 3.1, available at: BlastKOALA.

### Membrane lipid extraction

Lipids were extracted using a modified Folch extraction method. A volume of cells equal to 1 ml of 0.5 OD_600_ was collected by centrifugation (4000 r.p.m., 15 min, 4 °C). The supernatant was discarded and 50 µl lipid standard (prepared as a 10 µM stock), C_12_ Sphingosyl phosphatidylethanolamine (d17 : 1/12 : 0), was added giving a final concentration of 500 nM (Sigma-Aldrich). The pellet was then resuspended in 500 µl ice-cold methanol by vortexing. 300 µl sterile water was added to the cells, followed by 1 ml chloroform. The sample was vortexed for 30 s before being centrifuged at 5,000 r.p.m. for 15 min at 4 °C with the brake off. After this step, it was possible to see the separation of the upper methanol-water phase and lower hydrophobic chloroform phase. The lower phase was transferred to a fresh glass vial and the solvent was removed by evaporation overnight in the fume hood. Dried lipids were resuspended in 1 ml of acetonitrile: ammonium acetate (95 : 5, v/v). Samples were then analysed by LC-MS.

### LC-MS analysis for lipidomics

Lipid extracts were analysed by LC-MS using a Dionex Ultimate 3000-LC/MS (Bruker Compact Q-ToF, Bruker). Lipids were separated based on the polarity of their head group on a BEH Amide XP column (Waters). The column was maintained at 30 °C with a flow rate of 150 µl min^−1^. Each run consisted of a 10 min period of calibration with 95% acetonitrile and 5% 10 mM ammonium acetate (pH 9.2). After this, 3 µl sample was injected into the system and was run on a 30 min gradient of 5–28% 10 mM ammonium acetate (pH 9.2). Each sample was analysed in positive and negative ionization modes with autofragmentation.

## Results and discussion

### Phosphate starvation stimulates the production of alkaline phosphatase

To examine the changes in the proteome under Pi-limited conditions, *B. glumae* and *B. plantarii* were cultured overnight in LB medium and then resuspended in either phosphate-free MMA (−Pi) or MMA supplemented with 1 mM Pi (+Pi). After 24 h, samples were collected from both −Pi and +Pi cultures for proteomic analysis as well as to assess the alkaline phosphatase activity of the cultures. Genes encoding the alkaline phosphatases are part of the Pho regulon, which is activated by the PhoBR TCS under Pi-starvation conditions [31–33]. Both *B. plantarii* and *B. glumae* showed an increase in alkaline phosphatase activity at 24 h in −Pi condition (Fig. 1A). This increase in activity was not detected in +Pi cultures, which shows that phosphate starvation stimulates the production of alkaline phosphatase in both *B. glumae* and *B. plantarii,* as has been previously observed in other bacterial strains.

**Fig. 1.**
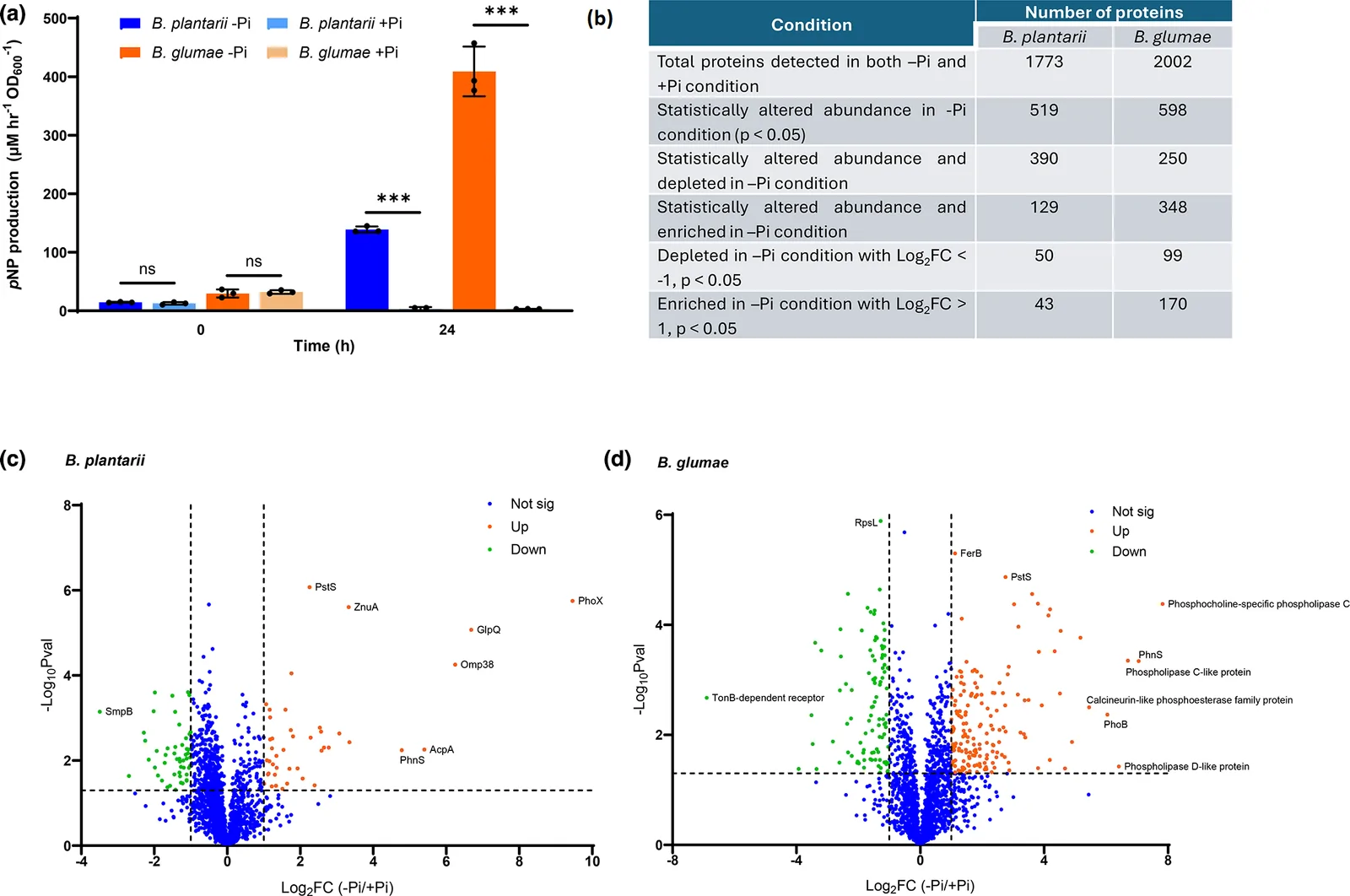
Proteomics analysis of *B. plantarii* and *B. glumae* in phosphate limitation. **(a**) Alkaline phosphatase activity of *B. plantarii* and *B. glumae*, cultured in the absence and presence of 1 mM Pi, was measured at 0 and 24 h by calculating the pNP production. Error bars represent the standard deviation of the mean calculated from three biological replicates, which are represented as individual data points. ns=non-significant, ***P<0.001 (Unpaired *t*-test, *P*-value was adjusted for multiple comparisons using Holm–Šídák method). (**b**) Summary of the total number of proteins which were detected in both −Pi and +Pi conditions and the number of proteins which had a significantly altered abundance (*P*<0.05) in *B. plantarii* and *B. glumae* cultured in −Pi condition for 24 h as compared to +Pi condition. (**c**) Volcano plots showing the log_2_FC of all the proteins that were detected when *B. plantarii,* and (**d**) *B. glumae* were cultured in −Pi condition for 24 h as compared to the +Pi condition. Proteins were considered to have a significantly altered abundance in −Pi condition relative to the +Pi condition if *P*-value was less than 0.05 (indicated by the horizontal dashed line). Vertical dashed lines represent a cut-off of 1 for log_2_FC with significantly enriched proteins (log_2_FC>1, *P*<0.05) shown in red and significantly depleted proteins (log_2_FC < −1, *P*<0.05) shown in green.

### Growth in phosphorus limitation condition induces significant changes in proteome

In order to compare the proteome of the cultures grown in −Pi and +Pi conditions, the samples collected after 24 h of growth were processed. The extracted proteomes were further analysed using LC-MS/MS. The correlation among three biological replicates from each condition (−Pi and +Pi) was checked by comparing the abundance of all detected proteins using XY plots (Fig. S1). Pearson’s correlation coefficient (*R*^2^) was over 0.8 for all the comparisons, indicating low variability and high reproducibility among replicates. Furthermore, in PCA plots, the replicates from the same condition were clustered together but there was a clear separation between samples from −Pi condition and +Pi condition (data not shown).

A total of 1,773 and 2,002 proteins were detected in *B. plantarii* and *B. glumae*, respectively (Fig. 1b, Tables S1 and S2), corresponding to a coverage of ~26% for *B. plantarii* and ~29% for *B. glumae*. Out of all the proteins detected, the number of proteins that had a significantly altered abundance (*P*<0.05) in −Pi condition as compared to the +Pi condition was 519 in *B. plantarii* and 598 in *B. glumae* (Fig. 1b).

Next, we investigated proteins that were highly enriched or depleted in −Pi condition (i.e. more than twofold changes). A total of 50 proteins were significantly depleted and 43 were significantly enriched under −Pi condition in *B. plantarii* (Fig. 1c). On the other hand, 99 proteins were significantly depleted and 170 were significantly enriched under −Pi condition in *B. glumae* (Fig. 1d). The top 10 most enriched and depleted proteins are shown in Table 1.

**Table 1.** Top 10 proteins which were either significantly enriched or depleted when cells were cultured in −Pi condition for 24 h as compared to the +Pi condition. All proteins have a *P*-value of less than 0.05

***B. plantarii***					
**Locus tag**	**Gene**	**Protein name**	**Protein function**	**Log**_**2**_ **FC**	**Reference**
***Enriched***					
Ga0123590_121070	*phoX*	Alkaline phosphatase PhoX	Removes phosphate groups from other molecules	9.46	[32]
Ga0123590_111999	*glpQ*	Glycerophosphoryl diester phosphodiesterase	Cleaves off glycerol-3-phosphate from phospholipids and wall teichoic acids under Pi starvation conditions	6.68	[45]
Ga0123590_112925	*omp38*	Predicted porin		6.24	
Ga0123590_121546	*acpA*	Acid phosphatase	Possibly related to Type 2 secretion system	5.40	[59]
Ga0123590_11535	*phnS*	2-Aminoethylphosphonate transport system substrate-binding protein	Part of the *phnRSTUVWX* operon which is induced under Pi starvation conditions. This cluster is involved in the transport and degradation of AEP.	4.78	[42]
Ga0123590_112657	*fabG*	3-Oxoacyl-[acyl-carrier protein] reductase	Fatty acid biosynthesis	3.35	
Ga0123590_123131	*znuA*	Zinc/manganese transport system substrate-binding protein	Binds zinc ions with high affinity allowing their transport across the membrane under zinc-limited conditions	3.33	[46–49]
Ga0123590_121820	*plc*	Phospholipase C	Breaks down phospholipids	3.07	[52]
Ga0123590_122670	*phaP*	Phasin family protein	PhaP proteins bind to the surface of PHA granules, which are storage structures for carbon and energy in certain bacteria. PhaP proteins can also function as chaperones to aid in protein folding.	2.78	
Ga0123590_1267	*atpE*	F-type H+-transporting ATPase subunit c	Encodes the c subunit of the ATP synthase	2.65	
***Depleted***					
Ga0123590_122503	*smpB*	SsrA-Binding protein	Facilitates the rescue of stalled ribosomes	−3.49	
Ga0123590_111435		Uncharacterized protein (DUF2252 family)		−2.70	
Ga0123590_111011		Iron complex outer membrane receptor protein	Involved in the transport of siderophore-iron complexes	−2.29	
Ga0123590_1178	*srpH*	Serine O-acetyltransferase	Helps in cysteine biosynthesis. SrpH is essential for sulphur assimilation in bacteria.	−2.25	
Ga0123590_111764		General bacterial porin, GBP family		−2.15	
Ga0123590_112538		Predicted porin		−2.02	
Ga0123590_121640		Hypothetical protein		−1.98	
Ga0123590_111747	*ladA*	Long-chain alkane monooxygenase	Degradation of long-chain alkanes. Shares 69% identity with PA2348 hypothetical protein from *P. aeruginosa* PAO1, which was strongly upregulated (>tenfold) in the absence of sulphate relative to growth with inorganic sulphate.	−1.98	[60]
Ga0123590_121073	*guaA*	guanosine monophosphate (GMP) synthase (glutamine-hydrolysing)	Synthesizes GMP. This gene is linked to virulence in many bacterial species. In *Burkholderia pseudomallei*, a SNP was found in this gene in highly virulent isolates.	−1.93	[61]
Ga0123590_121952	*ssuD*	Alkanesulfonate monooxygenase subunit	Metabolizes alkanesulfonates	−1.90	
***B. glumae***					
***Enriched***					
BGL336gr_05528		Phosphocholine-specific phospholipase C	Breaks down phosphatidylcholine (PC) into DAG and phosphocholine.	7.82	
BGL336gr_01201	*phnS*	AEP transport system substrate-binding protein	Part of the *phnRSTUVWX* operon which is induced under Pi starvation conditions. This cluster is involved in the transport and degradation of AEP.	7.05	[42]
BGL336gr_03269		Phospholipase C-like protein	Structurally similar to phospholipase C enzymes but lacks catalytic phospholipase C activity	6.71	
BGL336gr_06272		Phospholipase D-like protein	Possibly breaks down phospholipids like phospholipase D	6.41	
BGL336gr_05881	*phoB*	TCS, OmpR family, phosphate regulon response regulator PhoB	Positive regulator of the phosphate regulon which is involved in phosphate uptake and utilization	6.04	[13, 14]
BGL336gr_04586		Calcineurin-like phosphoesterase family protein	Metallophosphatases that can act on a variety of organic phosphate substrates, including phosphomonoesters, phosphodiesters and potentially nucleic acids, nucleotides or components of the cell surface matrix	5.45	
BGL336gr_01495		Phospholipase C-like protein	Structurally similar to phospholipase C enzymes but lacks catalytic phospholipase C activity	5.17	
BGL336gr_05519	*plc*_*1*_	Phospholipase C	breaks down phospholipids	4.90	[52]
BGL336gr_05523	*pstA*	Phosphate transport system permease protein	PstA encodes an integral membrane protein crucial for the phosphate-specific transport system PstSABC	4.67	[35–37, 41]
BGL336gr_05524	*pstB*	Phosphate transport system ATP-binding protein	PstB is the ATP-binding subunit of a high-affinity phosphate-specific uptake system, PstSABC	4.53	[35–37, 41]
***Depleted***					
BGL336gr_02448		TonB-dependent receptor	Uptake and transport of large substrates, such as iron siderophore complexes and vitamin B12	−6.90	
BGL336gr_06418	*mliC*	Membrane-bound inhibitor of C-type lysozyme	Inhibits the activity of C-type lysozymes, which are part of the animal innate immune system	−3.93	
BGL336gr_04460	*cydA*	bd-type cytochrome oxidase subunit I	Component of the bacterial respiratory chain	−3.51	
BGL336gr_04599		Acetoacetyl-CoA reductase	Key enzyme in the biosynthesis of the energy storage polymer, poly(3-hydroxybutyrate)	−3.47	
BGL336gr_02055		TonB-dependent receptor	Uptake and transport of large substrates, such as iron siderophore complexes and vitamin B12	−3.40	
BGL336gr_01260		Outer membrane porin		−3.35	
BGL336gr_06689	*BPSL0790*	SnoaL-like protein	Small polyketide cyclase involved in the biosynthesis of polyketide antibiotics	−3.20	
BGL336gr_03565	*cspA*	Cold shock protein	Crucial for the cold-shock response. CspA functions as an RNA chaperone to prevent mRNA from forming rigid secondary structures at low temperatures, which facilitates translation under cold stress. CspA can also function as a transcription antiterminator, preventing premature termination of transcription.	−2.82	
BGL336gr_02492		Large subunit ribosomal protein L3	Crucial for protein synthesis	−2.61	
BGL336gr_02868		Condensation domain-containing protein	Catalyse the formation of peptide bonds during non-ribosomal peptide synthesis	−2.60	

### Proteins associated with phosphate stress response are significantly enriched under Pi-limited condition

One of the most prominent responses to Pi limitation in both *B. plantarii* and *B. glumae* was the strong upregulation of transport systems involved in phosphate acquisition (Table 2). Components of the high-affinity phosphate-specific transport system PstSABC were significantly enriched in Pi-depleted cultures of both organisms. In *B. plantarii*, the substrate-binding protein PstS, the permease PstA and the ATP-binding subunit PstB showed increased abundance, whereas *B. glumae* displayed enrichment of all core components of the transporter, including PstS, PstA, PstB and PstC. The induction of the Pst system is a hallmark of phosphate starvation. It is typically under the control of the Pho regulon and has been well characterized in other soil bacteria, such as *Bacillus subtilis*, *Sinorhizobium meliloti*, *Bacillus licheniformis* and *Streptomyces coelicolor* [34–41]. Furthermore, PhoB and PhoR, which control the transcription of the Pho regulon, were also enriched under Pi-limited conditions in *B. glumae*. Interestingly, *B. glumae* also exhibited increased levels of PhoU, a negative regulator of the Pho regulon. Enrichment of PhoU under Pi limitation may reflect fine-tuning of phosphate homeostasis, preventing excessive activation of phosphate uptake systems once intracellular phosphate levels begin to recover.

**Table 2.** A selection of proteins that had a significantly enriched abundance after cells were cultured in −Pi condition for 24 h as compared to +Pi condition. Proteins with a |log2FC|≥ 2 and *P*<0.05 are shown.

***B. plantarii***					
**Locus tag**	**Gene**	**Protein name**	**Protein function**	**Log**_**2**_ **FC**	**Reference**
**Proteins detected in KEGG BRITE analysis**					
***Lipid biosynthesis proteins***					
Ga0123590_112657	*fabG*	3-oxoacyl-[acyl-carrier protein] reductase	Fatty acid biosynthesis	3.35	
***Transporters***					
Ga0123590_11535	*phnS*	AEP transport system substrate-binding protein	Phosphonate-binding protein	4.78	[42]
Ga0123590_123131	***znuA***	Zinc/manganese transport system substrate-binding protein	Binds zinc ions with high affinity allowing their transport across the membrane under zinc-limited conditions	3.33	[46, 49]
Ga0123590_121257	*pstB*	Phosphate transport system ATP-binding protein	PstB component of the high-affinity phosphate-specific uptake system PstSABC	2.58	[35–37, 41]
Ga0123590_121256	*pstA*	Phosphate transport system permease protein	PstA component of the high-affinity phosphate-specific uptake system PstSABC	2.28	
Ga0123590_121254	*pstS*	Phosphate transport system substrate-binding protein	PstS component of the high-affinity phosphate-specific uptake system PstSABC	2.25	
***Bacterial toxins***					
Ga0123590_121820	*plc*	Phospholipase C	Breaks down phospholipids	3.07	[52]
**Proteins not detected in KEGG Brite analysis**					
***Other proteins associated with phosphate stress***					
Ga0123590_121070	*phoX*	Alkaline phosphatase PhoX	Alkaline phosphatase	9.46	[32]
Ga0123590_111999	*glpQ*	Glycerophosphoryl diester phosphodiesterase	Phosphodiesterase	6.68	[45]
***Other virulence related proteins***					
Ga0123590_121546	*acpA*	Acid phosphatase	Acid phosphatase	5.40	[59]
Ga0123590_11652		Aryl-alcohol dehydrogenase-like predicted oxidoreductase	Predicted oxidoreductase	2.39	
***B. glumae***					
**Proteins detected in KEGG BRITE analysis**					
***Transporters***					
BGL336gr_01201	*phnS*	AEP transport system substrate-binding protein	Phosphonate-binding protein	7.05	[42]
BGL336gr_05523	*pstA*	Phosphate transport system permease protein	PstA component of the high affinity phosphate specific uptake system PstSABC	4.67	[35–37, 41]
BGL336gr_05524	*pstB*	Phosphate transport system ATP-binding protein	PstB component of the high-affinity phosphate-specific uptake system PstSABC	4.53	
BGL336gr_05522	*pstC*	Phosphate transport system permease protein	PstC component of the high affinity phosphate specific uptake system PstSABC	4.18	
BGL336gr_05883		Multidrug efflux pump	Removes toxic substances from the cell	3.37	
BGL336gr_01363		Putative spermidine/putrescine transport system substrate-binding protein	Polyamine transporter system	3.24	
BGL336gr_00875		NitT/TauT family transport system permease protein	ABC transporter that transports sulfonates and aas across cell membrane	2.86	[50]
BGL336gr_05521	*pstS*	Phosphate transport system substrate-binding protein	PstS component of the high affinity phosphate specific uptake system PstSABC	2.75	
***Secretion system***					
BGL336gr_06926	*traV*	Conjugal transfer pilus assembly protein TraV	Acts as an outer membrane anchor for a trans-envelope protein complex crucial for pilus assembly during bacterial conjugation	3.49	
BGL336gr_04999		Thioredoxin family protein		2.75	
BGL336gr_04724	*hcp*	Type VI secretion system secreted protein Hcp	Core component of the type VI secretion system (T6SS).	2.28	[62]
BGL336gr_02577	*tatC*	Sec-independent protein translocase protein TatC	Component of the sec-independent twin-arginine translocation (Tat) system that transports fully folded proteins across the cytoplasmic membrane.	2.12	
***Bacterial toxins***					
BGL336gr_05528		Phosphocholine-specific phospholipase C	Breaks down phosphatidylcholine (PC) into DAG and phosphocholine	7.82	
BGL336gr_03269		Phospholipase C-like protein	Putative phospholipase	6.71	
BGL336gr_06636		Phosphocholine-specific phospholipase C	Breaks down PC into DAG and phosphocholine	4.14	
BGL336gr_03183		Phospholipase C-like protein	Putative phospholipase	2.74	
BGL336gr_03196		Phospholipase C-like protein	Putative phospholipase	2.46	
BGL336gr_04464		Phosphocholine-specific phospholipase C	Breaks down PC into DAG and phosphocholine	2.15	
***TCS***					
BGL336gr_05881	*phoB*	TCS, OmpR family, phosphate regulon response regulator	PhoB response regulator	6.04	[13, 14]
BGL336gr_05882	*phoR*	TCS, OmpR family, phosphate regulon sensor histidine kinase	PhoR sensor kinase	3.61	[13, 14]
BGL336gr_05407	*cheY*	TCS, chemotaxis family, chemotaxis protein CheY	Regulates flagellar rotation and thus cell movement	2.30	
***Bacterial motility proteins***					
BGL336gr_05407	*cheY*	TCS, chemotaxis family, chemotaxis protein CheY	Regulates flagellar rotation and thus cell movement	2.30	
BGL336gr_05617	*mcp*	Methyl-accepting chemotaxis protein	Transmembrane receptor that detects chemicals in the environment to guide bacterial movement	2.15	
**Proteins not detected in KEGG Brite analysis**					
***Other proteins associated with phosphate stress***					
BGL336gr_04586		Calcineurin-like phosphoesterase family protein	Metallophosphatase that can act on a variety of organic phosphate substrates, including phosphomonoesters, phosphodiesters and potentially nucleic acids, nucleotides or components of the cell surface matrix	5.45	
BGL336gr_04439	*agt*	1,2-diacylglycerol 3-alpha-glucosyltransferase/glucuronosyltransferase	Glycolipid glycosyltransferase	4.51	[25]
BGL336gr_02277	*phoX*	Alkaline phosphatase	alkaline phosphatase	4.34	[32]
BGL336gr_02257	*ppk1*	Polyphosphate kinase	polyP synthesis	3.17	
BGL336gr_05880	*phoU*	Phosphate transport system protein	Negative regulator of Pho regulon	3.03	[21, 22]
BGL336gr_05325	*ppk2*	Polyphosphate kinase	Utilizes inorganic polyP to generate GTP from GDP. This function is crucial for bacterial stress tolerance, nucleotide pool regulation and virulence.	2.67	
***Other virulence related proteins***					
BGL336gr_06272		Phospholipase d-like protein	Putative phospholipase D	6.41	
BGL336gr_01495		Phospholipase C-like protein	Putative phospholipase	5.17	
BGL336gr_05519	*plc*_*1*_	Phospholipase C	Breaks down phospholipids	4.90	[52]
BGL336gr_03984	*acpA*_*1*_	Acid phosphatase	Acid phosphatase	4.19	[59]
BGL336gr_05364	*acpA*_*2*_	Acid phosphatase	Acid phosphatase	3.92	[59]
BGL336gr_02871	*cpdA*	3,5-cyclic-AMP phosphodiesterase	Hydrolyses cAMP to 5′-AMP. This function is crucial for regulating intracellular cAMP levels, which in turn affects various cellular processes, including catabolism, stress responses and biofilm formation.	2.68	[57]
BGL336gr_06432		Trimeric autotransporter adhesin	Virulence factor which plays roles in a wide range of biological processes, including adhesion, biofilm formation, host invasion and immune evasion	2.40	[54]
BGL336gr_03999		CheY-like protein	Possibly involved in bacterial chemotaxis	2.34	
BGL336gr_06608		Acyl transferase family protein	Performs O-acylation on various carbohydrates and other substrates, which can affect bacterial virulence and interactions with the host	2.34	[55]
BGL336gr_05406	*cheX*	Chemotaxis phosphatase CheX-like protein	Phosphatase that removes a phosphate group from the CheY response regulator	2.15	
BGL336gr_05531		Diguanylate cyclase	Cyclic diguanylate synthesis which regulates a wide range of cellular processes, including biofilm formation, motility, virulence and cell division	2.14	[56]
***General stress response***					
BGL336gr_05534	*rsbU*	Serine phosphatase RsbU	Regulates the general stress response pathway by activating the alternative sigma factor B (σ^B^)	3.39	[58]

In addition to Pi transport, both species showed strong enrichment of PhnS, the 2-aminoethylphosphonate (AEP) transport system substrate-binding protein. *phnS* is a part of the *phnRSTUVWX* operon which is induced under Pi starvation conditions [42]. This cluster is involved in the transport and degradation of AEP, which is a naturally occurring phosphonate present in great abundance in soil. The enrichment of PhnS suggests that both *Burkholderia* species enhance their capacity to acquire and utilize organophosphorus compounds when Pi becomes scarce. Utilization of organic phosphorus is common in other soil bacteria, such as *S. meliloti* and *Pseudomonas putida* [43, 44].

In *B. plantarii*, the alkaline phosphatases PhoX and PhoD were significantly enriched, with PhoX being the most enriched protein under Pi-limited conditions (Table 1). The second most enriched protein in *B. plantarii* was GlpQ, a glycerophosphoryl diester phosphodiesterase, which cleaves off glycerol-3-phosphate from phospholipids and cell wall teichoic acids during Pi-starvation [45]. Similarly, *B. glumae* displayed increased abundance of PhoX as well as a calcineurin-like phosphoesterase family protein (BGL336gr_04586) capable of hydrolysing a broad range of phosphomonoesters and phosphodiesters. These enzymes typically function to release Pi from diverse organic substrates present in the environment. The strong induction of these proteins, especially alkaline phosphatases and GlpQ, is consistent with previous studies showing that bacteria increase phosphatase activity during phosphate starvation to maximize phosphate recovery from organic matter [31–33, 45]. This mechanism is especially relevant in soil and plant-associated niches, where organic phosphorus often exceeds available Pi. Proteins involved in polyphosphate (polyP) metabolism were also consistently enriched under Pi limitation. Both species showed increased abundance of Ppk1, the enzyme responsible for polyP synthesis, facilitating Pi storage. In *B. glumae*, Ppk2, which breaks down polyP to release stored Pi, was also enriched.

### KEGG Brite enrichment analyses of differentially regulated proteins under Pi limitation

In addition to phosphorus stress response proteins discussed above, all proteins which had a |log_2_FC|>1 or < -1 and *P*<0.05 were analysed through the KEGG BRITE (Fig. 2, Tables S3 and S4). Under phosphorus-limiting conditions, the KEGG-defined proteome exhibits significant enrichment in metabolic enzymes and specialized transporters, reflecting a strategic shift toward phosphate scavenging and resource conservation. In addition, a large number of uncharacterized proteins also show high expression, suggesting untapped regulatory mechanisms in the Pi-starvation response. It is also worth noting that under Pi-stress conditions, several ribosome-associated proteins were significantly depleted in *B. glumae*’s proteome. A selection of proteins that were significantly enriched (*P*<0.05) and had a |log_2_FC|≥2 are discussed in the sections below (Table 2).

**Fig. 2.**
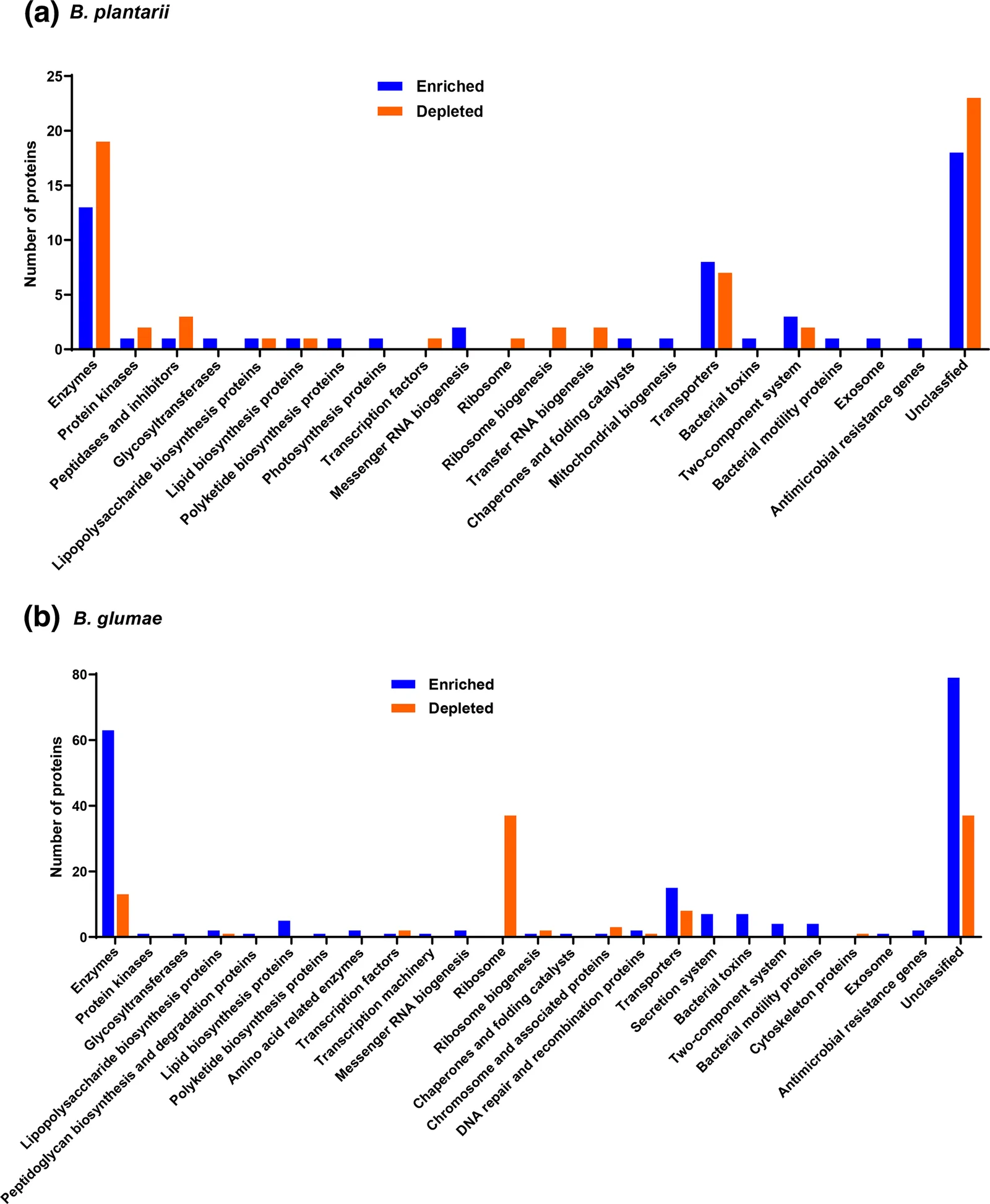
KEGG Brite analysis of differentially regulated proteins in phosphate limitation. Proteins with a significantly altered abundance (*P*<0.05) as well as |log_2_FC| > 1 or < -1, after (**a**) *B. plantarii* and (**b**) *B. glumae* were cultured in −Pi condition for 24 h relative to +Pi condition, were assigned to different KEGG BRITE categories using BlastKOALA (version 3.1).

#### Transporters

A wide range of transporters showed increased abundance under Pi limitation, reflecting enhanced scavenging of nutrients, expulsion of toxic compounds and upregulation of virulence factors. In addition to the PstSABC and PhnS transporters discussed above, *B. plantarii* showed enrichment of ZnuA, a high-affinity zinc-binding protein that imports zinc into the cells under zinc-limited conditions. ZnuA is commonly linked to virulence and *znuA* mutants are less virulent than their respective parent strain in many Gram-negative bacterial pathogens [46–49]. *B. glumae* showed enrichment of NitT/TauT family transport system permease protein, which transports sulfonates and aas across cell membranes. A homologue of this protein (BTH_I0896) is present in *Burkholderia thailandensis* E264 and is needed for meropenem (β-lactam) tolerance under carbon starvation conditions [50]. Furthermore, a multidrug efflux pump (BGL336gr_05883) was also enriched in *B. glumae*. Efflux pumps help cells expel antibiotics and toxic substances, including toxic host defence compounds. Establishing infection in a host usually requires overexpression and activation of efflux pumps. Additionally, *B. glumae* showed enrichment of a putative spermidine/putrescine transport system substrate-binding protein (BGL336gr_01363) which possibly helps the cells acquire nutrients from the environment during phosphate stress.

#### Bacterial toxins

Several toxin-related proteins were significantly enriched under Pi limitation. In both *B. plantarii* and *B. glumae*, phospholipase C and AcpA, an acid phosphatase that also exhibits phospholipase C activity, were enriched (Table 2). In *B. glumae*, multiple phosphocholine-specific phospholipase C proteins, phospholipase d-like proteins and phospholipase C-like proteins showed very strong enrichment, representing one of the most prominent responses observed. These enzymes can disrupt host cell membranes, release nutrients and promote bacterial dissemination [51, 52]. Their induction under Pi limitation suggests that phosphate stress may prime bacteria for aggressive nutrient acquisition from host-derived phospholipids.

#### Chemotaxis-associated proteins

Proteins involved in chemotaxis were consistently enriched under Pi-limited conditions. In *B. glumae*, the response regulator CheY, which is involved in bacterial chemotaxis, showed increased abundance. Furthermore, many other chemotaxis proteins, such as Mcp, which senses the chemical signals in the environment to regulate chemotaxis, a CheY-like protein which is possibly involved in chemotaxis, and phosphatase CheX which dephosphorylates CheY, were also enriched. The altered abundance of chemotaxis-associated proteins suggests that phosphate availability may influence chemotactic signalling. However, whether these proteomic changes translate into altered motility enabling bacteria to migrate towards more favourable niches or host tissues with higher nutrient availability remains to be determined experimentally. In *Pseudomonas* spp., specific methyl-accepting chemotaxis proteins have been implicated in phosphate sensing [53]. Although the functions of the MCPs identified here remain unknown, they represent interesting candidates for investigating phosphate-responsive chemotaxis in *Burkholderia*.

#### Other virulence-associated proteins

A diverse set of additional virulence-associated proteins was enriched under Pi-limited conditions. In *B. plantarii*, aryl-alcohol dehydrogenase-like predicted oxidoreductase (Ga0123590_11652), which possibly functions to detoxify xenobiotics, was enriched. In *B. glumae*, trimeric autotransporter adhesin (BGL336gr_06432), acyl transferase family protein (BGL336gr_06608), diguanylate cyclase (BGL336gr_05531) and 3,5-cyclic-AMP phosphodiesterase, CpdA (BGL336gr_02871) were enriched. Trimeric autotransporter adhesins play an important role in various aspects of pathogenesis, such as adhesion, biofilm formation and immune evasion [54]. Acyl transferase family proteins perform O-acylation on the carbohydrate component of the bacterial cell envelope, which can affect bacterial virulence and its interactions with the host [55]. Diguanylate cyclases synthesize the second messenger molecule cyclic diguanylate which regulates multiple virulence processes, including biofilm formation, adhesion, motility and virulence factor synthesis [56]. CpdA regulates intracellular cAMP levels, which in turn affects various cellular processes, such as stress responses and biofilm formation [57].

Proteomic profiling of *B. glumae* grown for 24 h under Pi limitation revealed significant enrichment of serine phosphatase RsbU. RsbU is a phosphatase which is part of the σ^B^-mediated general stress response. RsbU dephosphorylates pathway components to release the alternative sigma factor σ^B^, which allows σ^B^ to activate the general stress response. When bacterial cells are exposed to environmental stress or nutrient limitation, the general stress response protects various cell components, such as DNA, proteins and membranes against the oxidative stress. σ^B^-dependent general stress regulon is known to be activated under the conditions of phosphate stress in other soil bacteria, such as *B. subtilis* [58].

#### Lipid remodelling

Consistent with phosphate conservation strategies, proteins involved in lipid biosynthesis and remodelling were also enriched. The glycolipid glycosyltransferase Agt, which converts phospholipids to glycolipids under phosphate stress to fulfil the cellular Pi demand, was enriched in both species. This suggested both bacteria remodel their cell membrane during Pi stress.

### Lipidomics analysis reveals remodelling of the cell membrane during Pi stress

To examine whether cell membrane lipid changes occur during Pi stress, we cultured *B. plantarii* and *B. glumae* separately in the absence and presence of 1 mM Pi for 24 h and analysed their membrane lipid profile at the end of the growth using LC-MS. Both species produced glycolipids MGDG and GADG under Pi-deplete conditions at 24 h (Fig. 3). These glycolipids, however, were not present in Pi-replete conditions at 24 h or in samples taken at 0 h. These data show that both strains undergo extensive membrane remodelling under Pi limitation. Such remodelling reduces cellular phosphorus demand while potentially altering membrane properties linked to virulence and antimicrobial resistance [25, 28].

**Fig. 3.**
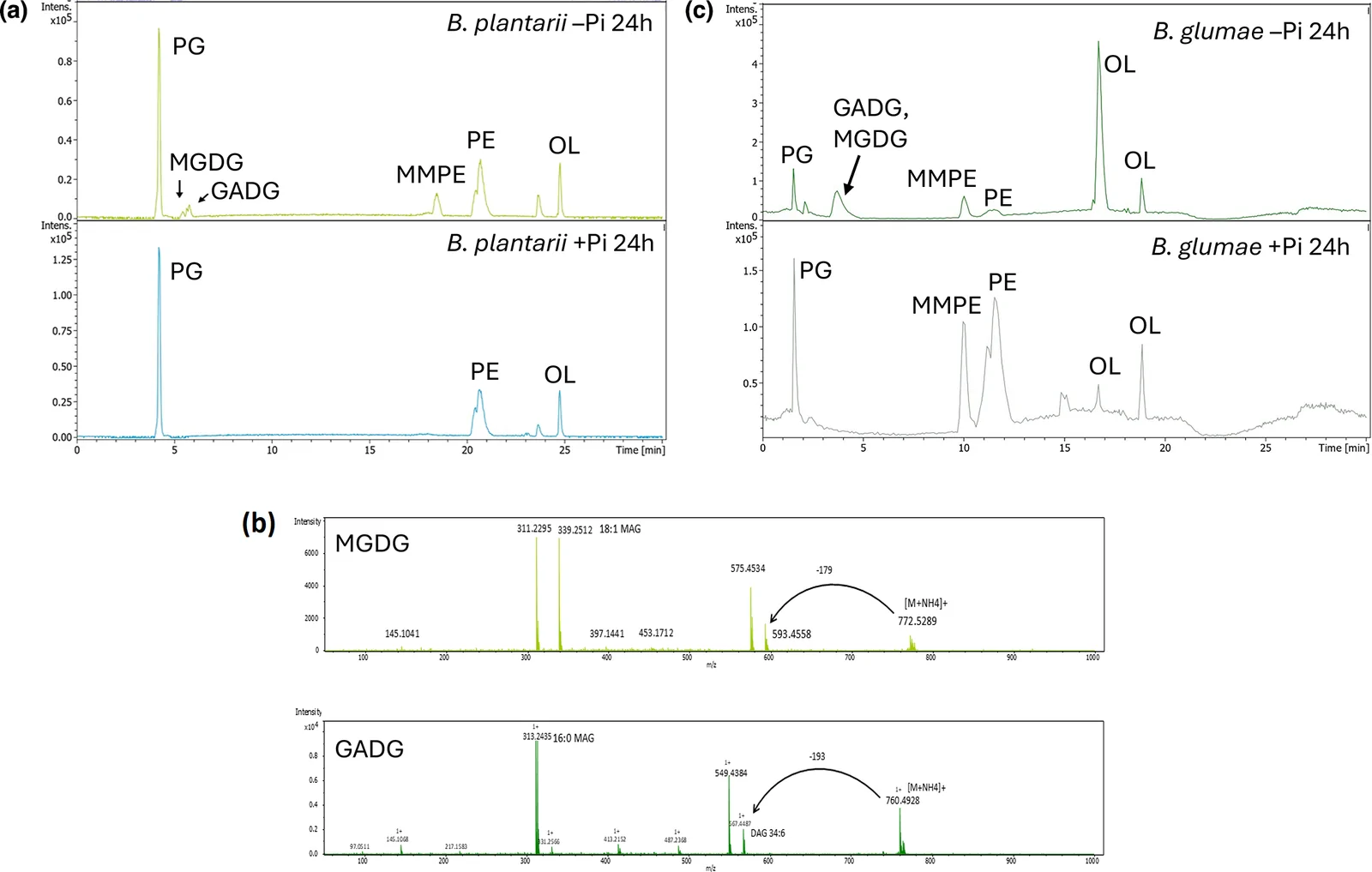
Formation of glycolipids in phosphate limitation. (**a**) Chromatograms showing the *B. plantarii* lipidome extracted from cultures grown in the absence and presence of Pi at 24 h. Peaks corresponding to glycolipids MGDG and GADG (shown with an arrow) were only observed in *B. plantarii* cultures grown in the absence of Pi for 24 h. Phosphatidylethanolamine, PE; Phosphatidylglycerol, PG; Monoglucosyldiacylglycerol, MGDG; Glucuronic acid diacylglycerol, GADG; Ornithine lipid, OL; monomethylphosphatidylethanolamine, MMPE. (**b**) Mass spectrometry fragmentation spectra of MGDG and GADG glycolipids observed in Pi-deplete *B. plantarii* cultures at retention times of 5.4 (m/z 772.5) and 5.8 (m/z 760.5) min, respectively. Each spectrum depicts an intact lipid mass with an ammonium (NH_4_^+^) adduct exhibiting neutral loss of a head group, yielding diacylglycerol (DAG) (shown with an arrow). Further fragmentation yields monoacylglycerols with C16 : 0 or C18 : 1 fatty acyl chains. (**c**) Chromatograms showing the *B. glumae* lipidome extracted from cultures grown in the absence and presence of Pi at 24 h. Peaks corresponding to glycolipids MGDG and GADG (shown with an arrow) were only observed in *B. glumae* cultures grown in the absence of Pi for 24 h.

## Conclusions

This work shows that Pi scarcity elicits extensive physiological reprogramming in *B. glumae* and *B. plantarii*, extending far beyond canonical phosphate-stress responses. Pi limitation stimulated strong induction of the Pho regulon, phosphonate uptake pathways and polyP metabolism, confirming activation of conserved phosphate-acquisition systems. Importantly, these changes were accompanied by increased abundance of proteins associated with virulence-related functions, including phospholipases, secretion systems, chemotaxis proteins and efflux pumps. The production of phosphorus-free glycolipids during Pi depletion further indicates major restructuring of the cell envelope to conserve phosphorus while potentially influencing host interaction and antimicrobial resistance.

Collectively, these findings demonstrate that phosphate availability is a key environmental determinant of pathogenic behaviour in *Burkholderia* phytopathogens. As soil phosphorus dynamics shift under changing agricultural and climatic conditions, understanding nutrient-responsive virulence mechanisms will be essential for predicting and managing the emergence and spread of *Burkholderia*-associated plant disease. The regulatory and metabolic pathways identified here provide a foundation for future work aimed at disrupting Pi-responsive virulence programmes to reduce disease impact.

## Abbreviations

ABC: ATP-binding cassette
AEP: 2-aminoethylphosphonate
DAG: diacylglycerol
GADG: glucuronic acid diacylglycerol
MGDG: monoglucosyl diacylglycerol
MMA: minimal medium A
PA: phosphatidic acid
PCA: principal component analysis
PE: phosphatidylethanolamine
PG: phosphatidylglycero
Pi: inorganic phosphate
pNP: *p*-nitrophenol
polyP: polyphosphate
SynCom: synthetic community
TCS: two-component system
TIMS-TOF: trapped ion mobility spectrometry with quadrupole time-of-flight
